# Impact of Regeneration on the Dosimetric Properties of LiF:Mg,Cu,P (MCP-N) Detectors

**DOI:** 10.3390/s26165176

**Published:** 2026-08-15

**Authors:** Patrycja Gordon, Katarzyna Matusiak, Mariusz Kłosowski, Aleksandra Jung

**Affiliations:** 1 Maria Skłodowska-Curie National Research Institute of Oncology, Kracow Branch, 30-059 Krakow, Poland; patrycja.m.gordon@gmail.com; 2 AGH University of Krakow, Faculty of Physics and Applied Computer Science, Department of Medical Physics and Biophysics, 30-059 Krakow, Poland; katarzyna.matusiak@fis.agh.edu.pl; 3 Institute of Nuclear Physics Polish Academy of Sciences, 31-342 Krakow, Poland; mariusz.klosowski@ifj.edu.pl

**Keywords:** thermoluminescent dosimetry, LiF:Mg,Cu,P (MCP-N) detectors, detector regeneration, glow-curve analysis, readout mode reproducibility

## Abstract

This study evaluates the dosimetric properties of standard (SD) and regenerated (RD) MCP-N thermoluminescent detectors over a wide dose range from mGy to Gy, with particular emphasis on the influence of readout mode, dose level, and temporal stability on detector performance. A total of 17 SD and 18 RD detectors were analyzed under standard conditions using two readout modes: a three-step READER mode and a continuous ANALYSER mode. Both detector types exhibit good linearity; however, deviations from ideal behavior at higher doses suggest the involvement of additional trapping and recombination processes. Glow-curve analysis indicates modifications in RD detectors, reflected by enhanced low-temperature peaks and slight shifts in peak position. Temporal effects also influence detector response, with stabilization observed after repeated irradiation–readout cycles. Fading remains low, with relatively stable signal levels over time. The results show that the ANALYSER mode provides improved stability and reproducibility compared to the READER mode, especially at higher doses, where increased variability and certain limitations were observed. Overall, regenerated detectors, despite reduced sensitivity, achieve performance comparable to standard detectors after stabilization. Optimization of readout conditions, particularly the use of ANALYSER mode, is essential for ensuring reliable dosimetric performance across a wide dose range.

## 1. Introduction

The application of ionizing radiation in medicine, industry, and scientific research requires rigorous control and continuous monitoring to ensure safety and compliance with regulatory standards. Accurate dosimetry is essential for occupational exposure assessment and for environmental radiation surveillance [1,2,3]. Apart from sensitive active dosimeters, such as semiconductor detectors or ionization chambers, so-called passive detectors are often used. They operate without an external power supply, making them particularly advantageous in long-term and distributed monitoring scenarios. Among these, thermoluminescent (TL) detectors represent one of the most widely used and reliable solutions. Their key advantages include small size, robustness, and the ability to integrate dose over extended periods. These features enable their application in personal dosimetry, where individual radiation exposure of workers can be assessed, as well as in environmental monitoring, including measurements in workplaces, natural environments such as caves, and areas surrounding nuclear facilities [4,5,6,7,8,9,10].

The compact dimensions of TL detectors further allow their deployment in specialized contexts where space is limited or accessibility is constrained. Notably, they have been successfully utilized in space missions, for example in anthropomorphic phantoms such as the Matroshka experiment, where detectors were embedded to evaluate radiation doses in conditions simulating human tissues [11,12]. Such applications highlight the versatility of passive dosimetry systems across a wide range of radiation fields.

The performance of thermoluminescent detectors strongly depends on the material composition and the presence of dopants, which influence trapping and recombination processes. Various materials have been developed and optimized for dosimetric purposes, including LiF:Mg,Ti (MTS-N), CaSO_4_:Dy [13], Al_2_O_3_:C, and LiF:Mg,Cu,P (MCP-N) [14,15,16,17]. Among these, MCP-N detectors are recognized for their exceptionally high sensitivity, often exceeding that of other commonly used TL materials by an order of magnitude. This makes them particularly suitable for low-dose measurements, such as in environmental monitoring or medical applications involving low radiation levels [16,18]. They exhibit good readout repeatability and are considered tissue-equivalent materials, showing sensitivity to UV-VIS light and to pre- and post-exposure annealing conditions, as well as the ability to detect any type of radiation, although with different efficiency. MCP-N detectors are characterized by a linear–sublinear response [1,2,11,19,20,21,22,23,24]. At short intervals, the amplitude of the low-temperature peaks decreases, while the amplitude of the dosimetric peak remains unchanged [12,25,26]. However, some properties, such as sensitivity, may decrease over time, requiring detector replacement. Therefore, in addition to the continuous search for and development of new materials, attempts are also made to improve the properties of already known materials, as well as to regenerate used detectors.

In recent years, increasing attention has been paid to the potential for reuse and recycling of dosimetric materials, motivated by both economic and environmental considerations and the growing importance of sustainable resource management [23,27,28]. The recovery and reuse of raw materials used in dosimetric systems may provide a more resource-efficient approach to detector production. Since LiF-based thermoluminescent materials have been extensively studied and widely applied in radiation dosimetry for decades, they constitute a well-characterized and reliable group of detectors. However, despite their long-term use and well-established properties, studies focused on the regeneration and reuse of LiF:Mg,Cu,P materials remain limited. Preliminary observations obtained during this work indicated that partially regenerated detectors may exhibit additional interesting properties beyond the environmental and economic benefits associated with material recovery, which motivated a detailed comparative analysis of regenerated and standard detectors. However, repeated irradiation and readout cycle may affect detector properties, such as sensitivity, fading characteristics, and reproducibility, necessitating careful evaluation and comparison of varied materials.

With reference to this research trend, the aim of the work was to characterize the dosimetric properties of partially regenerated detectors and compare them to reference detectors, namely standard detectors. As part of the work, the sensitivity, repeatability of readout and individual response factor, linearity of the dose characteristic and signal decay over time were assessed. Measurements were conducted in the low and high dose range, in two reading modes.

## 2. Materials and Methods

The study used seventeen standard and eighteen regenerated LiF:Mg,Cu,P detectors (so-called MCP-N detectors, manufactured by IFJ PAN, PL-31342 Krakow, Poland) in the form of round pellets with a diameter of 4.5 mm and a thickness of 0.9 mm. The standard detectors were new and produced in the standard cycle described earlier [28], except that the phosphorus-doping substance (NH_4_)_2_PO_4_ was replaced by H_3_PO_4_. The doping concentrations in the standard detectors were 0.2 mol% Mg, 0.05 mol% Cu, and 1.25 mol% P. The regenerated detectors were manufactured using the same technological cycle but from different input material. The melt batch consisted of two powders mixed in a 1:1 weight ratio. One half was lithium fluoride with the addition of dopants at concentrations of 0.2 mol% Mg, 0.05 mol% Cu, and 1.25 mol% P (identical to the standard doping). The other half was LiF powder recovered from detector production waste. LiF recovery involved removing impurities from grains smaller than 63 μm using 1 M HCl; the hydrochloric acid removed Cu^2+^ ions. The acid-washing process was repeated several times until the blue coloration from Cu^2+^ ions was eliminated. After drying the recovered LiF, the dopants were reintroduced. The impurity concentrations in the regenerated detectors were 0.2–0.3 mol% Mg, 0.05 mol% Cu, and 1.25 mol% P.

The dopants concentrations were calculated based on the initial composition of the raw materials and the amounts of added dopants. It was assumed that copper ions would be completely removed from the regenerated material, and that the magnesium ion concentration would be within the limits consistent with the removal of magnesium ions and retention in the structure of the regenerated material. Due to the size of the PO_4_^3−^ ion, it is expected that this ion will not leach from the regenerated material. Direct analytical confirmation of the actual admixture content was not performed, which is considered a limitation of this study.

The annealing and readout procedure was performed in a repeatable manner and adapted to the LiF: Mg, Cu, P material. Pre-exposure and post-exposure heating was conducted using dedicated TLDO oven (produced by PTW). The detectors were annealed at 240 °C for 10 min prior to radiation exposure. Irradiation was carried out using the Cs-137 source for low doses and a medical linear accelerator for high doses irradiation. After irradiation, the detectors underwent post-irradiation annealing at 100 °C for 10 min. The readout procedure was performed using the Laboratory Reader—Analyzer RA’04 by Microlab. The detectors were read from the unmarked side. In the ANALYSER mode, the temperature increased linearly to a maximum temperature of 245 °C with a heating rate during the reading of 2 °C/s. In READER mode, the temperatures were increased in 3 stages: 150 °C for 10 s, 245 °C for 15 s and, finally, 245 °C for 10 s [29]. Both READER and ANALYSER modes were applied to every detector type and dose level, allowing the two variables to be evaluated independently rather than confounded: a difference between standard and regenerated detectors that is consistent across both readout modes can be attributed to the detector itself, whereas a difference specific to one mode reflects a readout-method artifact. READER mode offers faster throughput via simplified signal integration, while ANALYSER mode evaluates the full glow curve shape and is therefore less sensitive to shape-related artifacts at high doses, consistent with established practice for MCP-N detectors [3,30,31].

Between readings, the detectors were protected against UV-VIS light. Results were expressed as the number of counts.

The division of detectors according to the type and mode of readout is presented in Table 1.

Homogeneity, sensitivity, repeatability of detector reading, stability of readout in the group, stability of the individual response factor, linearity of the dose characteristic for various dose ranges and fading were assessed in the study.

Detector **homogeneity** is a property that applies to a group of detectors. In order to obtain information on the homogeneity of the detectors, the so-called zero measurement (i.e., without prior exposure) was performed. Before the measurement, the detectors were annealed at 240 °C. The homogeneity was calculated based on Equation (1).

(1) $${∆}_{max}=\frac{{\left(x-x_{0}\right)}_{max}-{\left(x-x_{0}\right)}_{min}}{{\left(x-x_{0}\right)}_{min}}$$

where

- x—value of the number of counts from the last measurement series in which the detectors were irradiated with the dose of 1 mGy.
- $x_{0}$—value of the number of counts from the so-called zero measurement [16,26,32].

An **individual response factor** (IRF) was calculated for each detector based on reference measurements in which the entire group of dosimeters received the same dose. In the study, the individual response factor was determined for the dose of 1 mGy, 4 mGy and 0.24 Gy. For the i-th detector, it was calculated as the ratio of the average value of the number of counts of the entire group $\bar{X}$ to the value of the number of counts of a given detector $X_{i}$ (Equation (2)) [3,32].

(2) $${IRF}_{i}=\frac{\bar{X}}{X_{i}}$$

**Sensitivity** is the basic parameter that characterizes thermoluminescent detectors. It is defined as the ratio of the TL signal to the dose absorbed. The sensitivity is determined by such factors as: detector base material, type of admixtures or detector form [4].

To assess the sensitivity of a detector made of a new material, the relative sensitivity is calculated, defined as the sensitivity of the new material in relation to the sensitivity of the reference detector. Both detectors should be subjected to the same conditions of irradiation, annealing and reading. It is also possible to observe how sensitivity changes during the following measurements with respect to the first measurement in the series [4,16,30].

**Repeatability R** is the ability to maintain a stable detector response to a selected dose. It can be assigned individually to a single detector or a group of detectors. The repeatability of R was calculated according to Equation (3).

(3) $$R=\left(1-\frac{u\left(M\right)}{M}\;\right)*100\;[\%]$$

where

M—the median detector response calculated from multiple measurement series;

u(M)—uncertainty of median [16,30,33].

The ability to obtain uniform detector responses to the selected dose was defined as the **stability of the readings in the group R_s_**. In the study the stability of detector responses for doses of 0.3 Gy, 1 Gy and 2 Gy was assessed. During readings in READER mode for higher dose values, low stability of the readings was noticed, therefore, the usefulness of the use of mechanical filters was additionally verified. The stability of the reading $R_{S}$ was determined analogously to the repeatability, with the median being determined based on the readings of several detectors corrected by the IRF. The $R_{S}$ parameter was determined according to Equation (4).

(4) $$R_{S}=\left(1-\frac{u\left(M\right)}{M}\;\right)*100\;[\%]$$

where

M—the median response of a detector group for a given dose, determined for a selected dose in a single measurement series;

u(M)—uncertainty of median.

The **linearity** of detector responses to a dose of ionizing radiation was assessed in the low dose ranges (1–4 mGy, 3 series) and in the high dose ranges (0.5–2 Gy). Two series of measurements were performed in the high dose ranges, in which a different division of the detectors was used: in the first series, the detectors were divided into 2 groups, while in the second series—into 4 groups [16,30,32].

The **fading** study was carried out in terms of different time intervals between the day of irradiation of the detectors and the day of read out. Signal loss was calculated as in [30]. The fading study was performed using the same set of MCP-N detectors employed in the ANALYSER-mode measurements reported elsewhere in this study, with the same number of detectors per group (Table 1). Since TL readout is destructive, each detector was annealed, re-irradiated, and individually read out for each storage interval; the resulting signal was normalized to that detector’s own most recent reference reading, thereby accounting for individual sensitivity variation between measurement cycles rather than relying on a group average.

The analysis was quantitative in the case of the READER mode, while in the ANALYSER mode, both quantitative (area under the curve) and qualitative (based on the shape of the TL curves) analyses were performed.

The reading of each detector was normalized using the IRF determined in the reference measurements preceding the actual test when it was necessary.

Results were presented as the median value with range (minimum–maximum), unless stated otherwise. For a single reading, the uncertainty was calculated from counting statistics as the square root of the number of counts. The relative uncertainty for each dose point was calculated as the ratio of the range to the median.

## 3. Results

### 3.1. Dose Response

**Figure 1:**
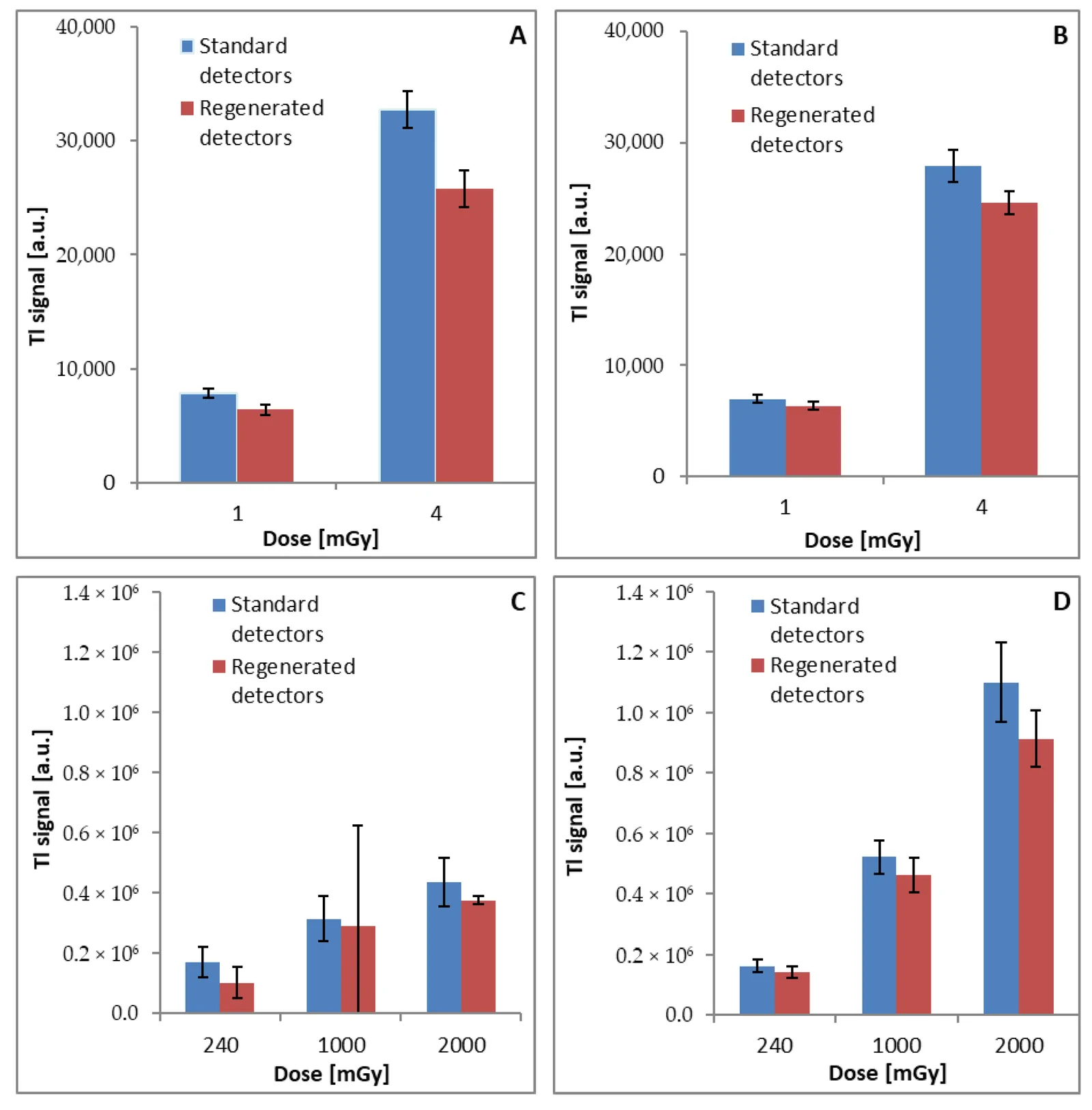
Comparison of the median response of standard and regenerated detectors for low-dose (upper panels: (**A**)—READER mode, (**B**)—ANALYSER mode) and high-dose ranges (lower panels: (**C**)—READER mode, (**D**)—ANALYSER mode).

The median dose response for both types of detectors was determined based on measurements obtained from six series at an irradiation dose of 1 mGy, four series at 4 mGy, two series at 0.24 Gy, and single irradiations at 1 Gy and 2 Gy. The associated uncertainty was estimated based on the observed range of values. The results are presented graphically in Figure 1.

The contribution of the signal from regenerated detectors relative to standard ones showed a decreasing trend with increasing dose, amounting to 81.0%, 78.7%, and 60.6% for 1 mGy, 4 mGy, and 0.24 Gy, respectively, in READER mode, and to 91.4%, 88.1%, and 85.6% in ANALYSER mode. This trend indicates that regenerated detectors exhibit progressively lower sensitivity as the irradiation dose increases. Furthermore, the regenerated detectors consistently demonstrate higher relative signal contributions in ANALYSER mode compared to READER mode, suggesting improved performance under ANALYSER readout conditions.

The relative uncertainties in ANALYSER mode remained low and stable across all doses, typically within ~3.9–10.0% for both standard and regenerated detectors. In contrast, READER mode shows comparable uncertainties at low doses (~4–6%) but exhibits pronounced outliers for regenerated detectors at intermediate and higher doses, reaching up to 51.8% and 116.8%, indicating substantially reduced measurement stability under these conditions. This pattern is consistent with the Reader’s saturation threshold, located at approximately 1 Gy: readings around this dose approach the limit of reliable photomultiplier response, which disproportionately amplifies the uncertainty (Figure 1C), whereas the 2 Gy measurement already exceeds this threshold, yielding signals below the level expected from linear extrapolation and, consequently, more closely clustered—and therefore less variable—results. Notably, although standard detectors generally exhibit lower variability, regenerated detectors show a smaller spread at selected dose points, such as 4 mGy and 2 Gy in ANALYSER mode.

Taken together, the results indicate that regenerated detectors exhibit reduced sensitivity with increasing dose, accompanied by a pronounced rise in measurement uncertainty in READER mode. In contrast, ANALYSER mode ensures more stable performance, maintaining relatively higher signal contributions and consistently low uncertainties, which highlights its advantage for reliable measurements of regenerated detectors. It should also be noted that the number of measurement series varied across dose levels (ranging from multiple series at low doses to single irradiations at the highest doses), which may have additionally influenced the observed variability and contributed to the uncertainty estimates derived from the range of values.

In ANALYSER mode, the shape of the thermoluminescence glow curves was additionally evaluated. For standard detectors, the curves are highly consistent, showing minimal variation regardless of detector number or measurement series within the cycle. In contrast, regenerated detectors exhibit a greater dispersion of curves, with the average amplitude associated with higher uncertainty. The peak maximum for regenerated detectors is shifted toward lower temperatures compared to standard detectors (approximately 213 °C vs. 218 °C) and is characterized by a lower amplitude (approximately 210 vs. 270, respectively). At the same time, the glow peak for regenerated detectors is broader, with a larger full width at half maximum (approximately 25 °C compared to 20 °C for standard detectors). Furthermore, the spread in peak position is slightly greater for regenerated detectors. The relative uncertainty of the peak position remains low for both detector types, amounting to approximately 1% for standard and regenerated detectors.

### 3.2. Homogeneity

In order to assess batch homogeneity, the homogeneity parameter was calculated based on the results obtained in the so-called zero measurement (i.e., without irradiation) and the results obtained in the twelve series at an irradiation dose of 1 mGy (Table 1). The group of detectors can be considered homogeneous if the value of the determined parameter does not exceed the limit of 30% [16].

Based on the results summarized in Table 2, it can be concluded that three out of four groups of detectors meet the homogeneity criterion with very good results (7–8%), except for regenerated detectors measured in READER mode, for which the homogeneity parameter reaches approximately 20%, i.e., more than twice the typical values observed for the other groups. This observation is, however, consistent with the previously discussed results. When the homogeneity parameter is determined based on the dosimetric peak amplitude, slightly better values are obtained for regenerated detectors. In contrast, when the parameter is conventionally calculated based on the area under the glow curve, regenerated detectors exhibit slightly lower values than standard ones.

### 3.3. Change in Sensitivity over Time

**Figure 2:**
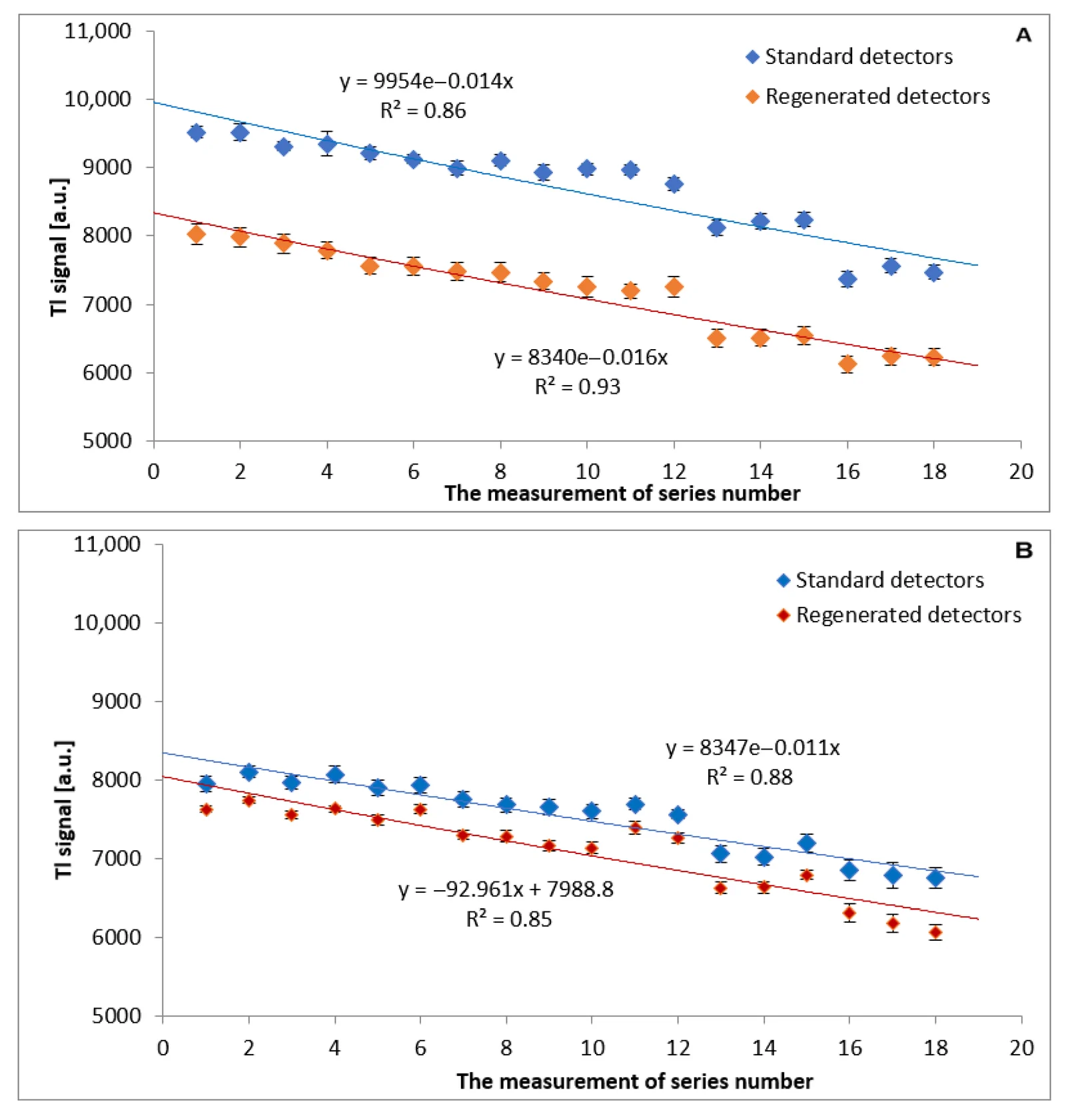
Change in detector average sensitivity over time during repeated irradiations at a dose of 1 mGy: READER mode (upper graph, (**A**)) and ANALYSER mode (lower graph, (**B**)).

**Figure 3:**
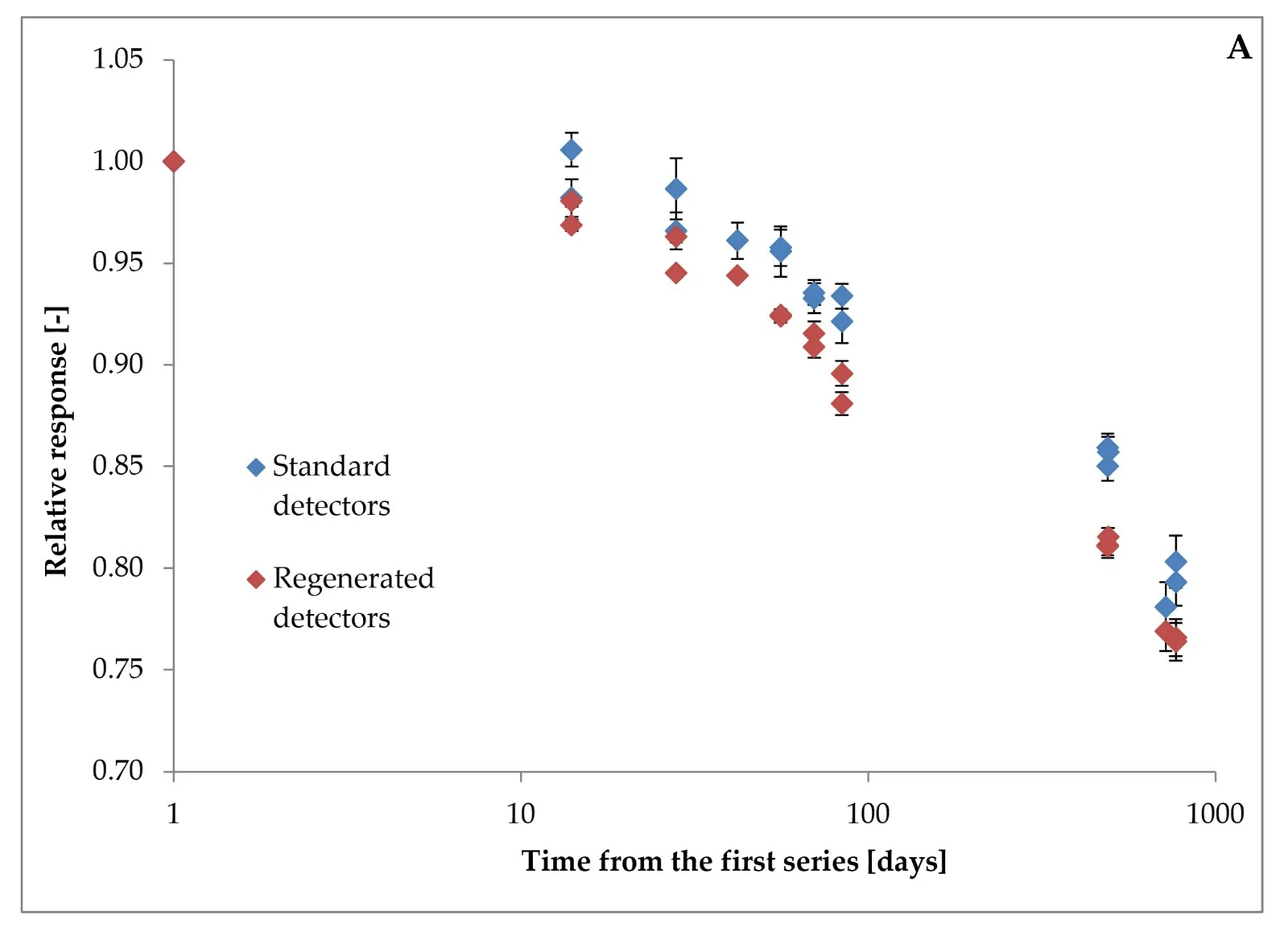

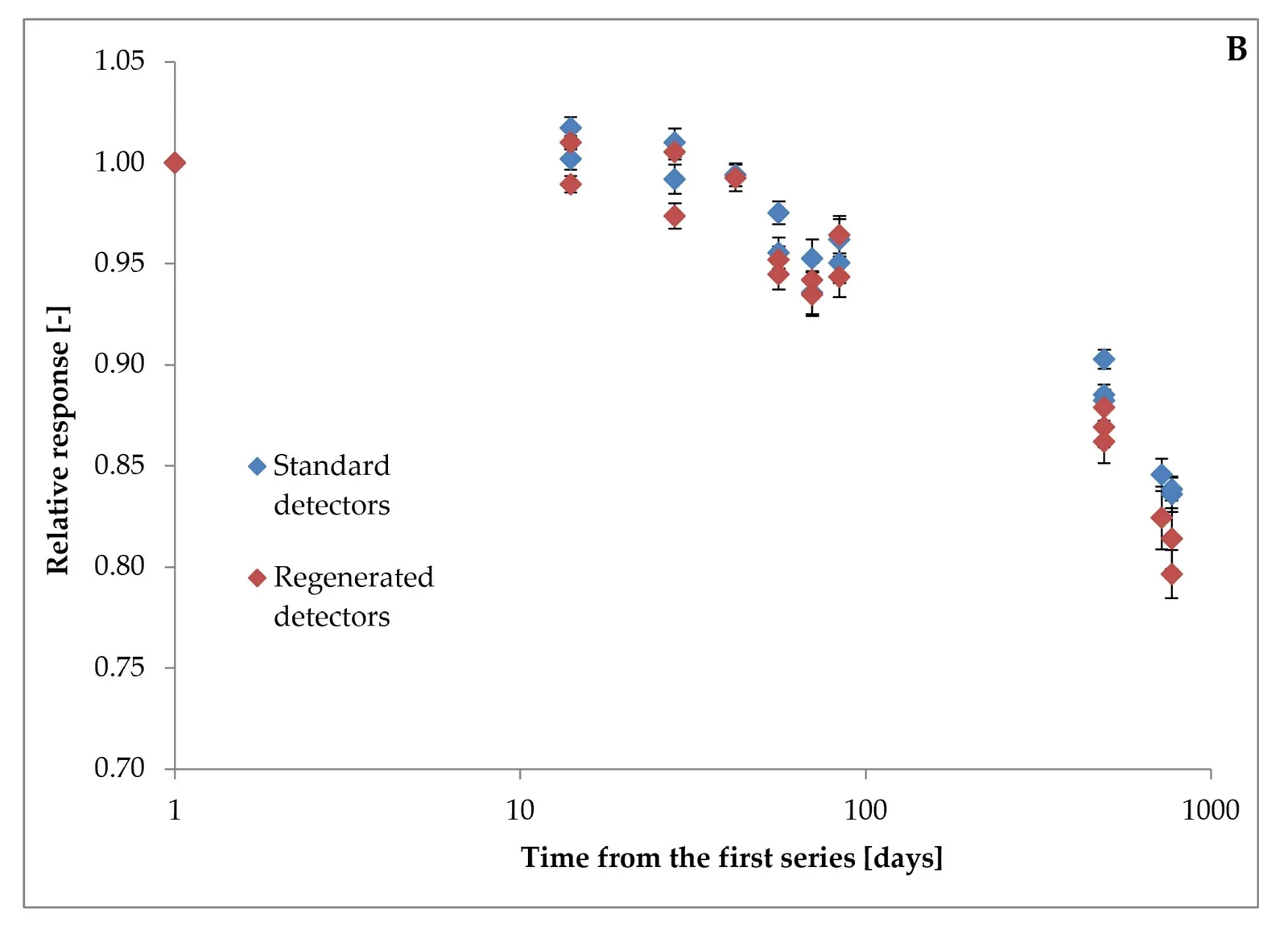
Detector readings normalized to the first measurement: READER mode (upper panel, (**A**)) and ANALYSER mode (lower panel, (**B**)).

Due to the tendency of MCP-N detectors to lose sensitivity over time, especially during the initial readouts, the detector response to a low dose of 1 mGy was evaluated based on 18 irradiation series at this dose. The focus on low-dose measurements reflects their practical importance, as such dose levels are most encountered in individual and environmental dosimetry. In Figure 2 and Figure 3 the detector dose response over 18 series is presented, with Figure 2 showing the absolute readings and Figure 3 showing the values normalized to the first measurement for successive days following the initial irradiation.

### 3.4. Repeatability

In this study, the reproducibility of the readout for individual detectors (Table 3) was evaluated for doses of 1 mGy, 4 mGy, and 0.24 Gy. Particular attention was given to the 1 mGy dose, for which a total of 18 measurement series were performed to investigate temporal changes in repeatability. A noticeable shift in detector sensitivity was observed after a prolonged time interval between the 15th and 16th series, indicating the influence of aging effects or storage conditions on detector response. Consequently, the results are presented both for all 18 series and separately for the last three series, which demonstrate improved stability and suggest partial recovery or stabilization of detector properties over time although this stabilization was accompanied by a gradual decrease in sensitivity. For the remaining two doses, the reproducibility was evaluated based on three measurement series.

A clear dependence of reproducibility on both dose level and readout mode can be observed. For the lowest dose (1 mGy), the initial measurement series (1–18) are characterized by relatively high variability in both READER and ANALYSER modes, with relative uncertainties exceeding 9–14%. However, a substantial improvement in repeatability is evident when only the last three series (16–18) are considered, where the relative uncertainty decreases to approximately 1.6–4.3%. This confirms that detector response stabilizes over time after an initial period of sensitivity changes.

For the intermediate dose (4 mGy), the reproducibility is better and remains stable regardless of the detector type or readout mode, with relative uncertainties below ~3%, indicating that temporal effects are less pronounced at this dose level. In contrast, for the high dose (240 mGy), a strong divergence between readout modes is observed. In READER mode, extremely high variability occurs, with relative uncertainties exceeding 100%, reflecting severe instability and poor reliability of measurements under these conditions. On the other hand, ANALYSER mode maintains relatively low uncertainties (approximately 5–9%), demonstrating its robustness even at higher doses.

When comparing detector types, standard detectors generally exhibit slightly better reproducibility, particularly in READER mode and at lower doses, where they tend to show lower relative uncertainties than regenerated detectors. However, after the stabilization period (series 16–18), the differences between standard and regenerated detectors become small. For the 1 mGy dose in READER mode, regenerated detectors show marginally lower relative uncertainty (1.6%) compared to standard detectors (2.7%), indicating slightly better repeatability under stabilized conditions. A similar tendency is observed in ANALYSER mode, where after stabilization the results for regenerated detectors become fully comparable to those of standard detectors. Overall, regenerated detectors can achieve equal or even slightly improved reproducibility once their response has stabilized over time.

Overall, these results indicate that both detector stabilization over time and the choice of readout mode play a critical role in determining measurement reproducibility. ANALYSER mode provides consistently more reliable and stable results across all dose ranges, while READER mode is strongly affected by both initial sensitivity changes and high-dose instability.

Additional analysis of the individual response factors (IRFs) shows trends analogous to those observed for repeatability. For the dose of 4 mGy, IRF values are highly consistent and close to unity (99.7–99.9%), with very low variability (0.24–0.49%), indicating excellent stability regardless of detector type or readout mode. In contrast, for the high dose (240 mGy), a strong divergence between readout modes is observed. READER mode is characterized by a very large spread of IRF values (approximately 75.7–84.9% with ranges up to ±48.7–53.4% and relative uncertainties of ~62–64%), confirming its instability at higher doses. In contrast, ANALYSER mode maintains narrow distributions (approximately 98.8–98.9% with ranges ±2.1–2.9% and relative uncertainties of ~2–3%), demonstrating high robustness.

Overall, standard detectors tend to exhibit slightly lower variability, particularly in less stable conditions; however, under stabilized conditions and in ANALYSER mode, regenerated detectors achieve comparable performance. These findings are consistent with previous observations, highlighting the dominant influence of readout mode and temporal stabilization on detector response.

### 3.5. Stability of the Readings

For high doses (1 and 2 Gy), repeated irradiations across multiple series were limited; therefore, the stability of the detector group response at these dose levels was evaluated to verify the reliability of measurements under these conditions. The stability of the readings was evaluated for doses of 1 Gy and 2 Gy. Table 4 summarizes the values of the reading stability parameter. The individual detector responses were normalized using IRF values determined during reference measurements performed immediately prior to the stability assessment.

In READER mode, stable readout behavior was not observed for either 1 Gy or 2 Gy. In the case of regenerated detectors at 2 Gy, the R_s_ parameter even reaches a negative value, reflecting extremely high variability, with the median uncertainty exceeding 100%. In contrast, in ANALYSER mode, the stability parameter remains high, with values above 90% for both doses.

For a dose of 1 Gy, regenerated detectors exhibit slightly better stability compared to standard detectors, whereas for 2 Gy the opposite trend is observed, with standard detectors showing superior performance. However, due to the limited number of measurements at these dose levels, these results should be treated with caution and require further verification to confirm their reproducibility.

To verify whether mechanical filtration (i.e., narrowing of the aperture during readout) can improve measurement stability, a short test was performed for a dose in the higher dose range (0.3 Gy). The results confirmed the previously observed instability of the READER mode at elevated doses, while demonstrating that the application of filtration can enhance the repeatability of the readings. This suggests that proper configuration of the readout system, particularly for higher dose levels, may substantially improve measurement stability in READER mode.

### 3.6. Linearity of Dose Response

**Figure 4:**
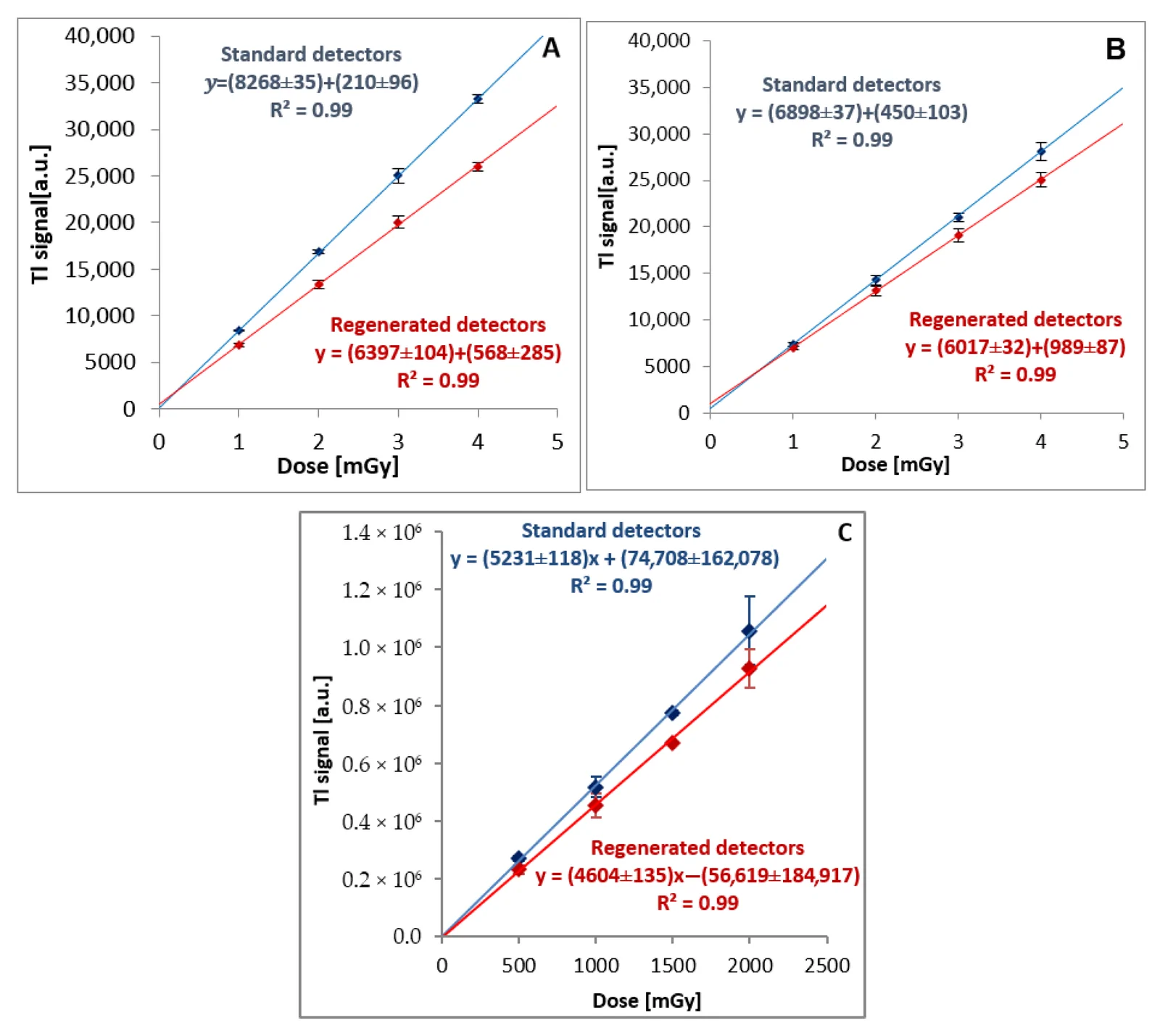
Dose–response characteristics of standard and regenerated detectors in the 1–4 mGy range (upper panels: (**A**)—READER mode, (**B**)—ANALYSER mode) and 0.5–2 Gy range (lower panel: (**C**)—ANALYSER mode). For low doses, the plotted values represent the median detector response obtained from three measurement series, whereas for high doses they correspond to the median response from a single measurement series.

The linearity of the dose–response characteristics of standard and regenerated detectors was assessed in two dose ranges: 1–4 mGy and 0.5–2 Gy (Figure 4). For low doses, the response in READER mode is presented as median counts for individual detectors, while in ANALYSER mode it is expressed as the area under the glow curve. For higher doses, results are shown exclusively for ANALYSER mode due to the previously demonstrated instability of the READER readout under these conditions.

The obtained results were normalized using IRF values determined in reference measurements preceding this study. For each dose, the median detector response was calculated from three measurement series, while the uncertainty was estimated based on the range of the readings. A linear function of the form y = ax + b was fitted to the experimental data.

The fitted linear functions demonstrate good agreement with the experimental data in the low-dose range (1–4 mGy), characterized by well-defined slopes and relatively small uncertainties in both READER and ANALYSER modes. The relative uncertainties of the slope parameter remain low, amounting to approximately 0.4–1.6% (READER mode) and ~0.5% (ANALYSER mode), indicating reliable linear behavior. In this range, both standard and regenerated detectors exhibit consistent linearity, although slightly higher uncertainties and lower sensitivity are observed for regenerated detectors in READER mode, indicating marginally reduced precision compared to standard detectors.

For the higher dose range (0.5–2.0 Gy) only the results obtained in ANALYSER mode are presented and discussed, owing to the limitations of the READER mode described above. The linear fit parameters exhibit larger uncertainties, with relative uncertainties of the slope reaching approximately 2.3% for standard detectors and 2.9% for regenerated detectors. Additionally, very large uncertainties associated with the intercept term (b) are observed, indicating reduced reliability of the fit. This effect is more pronounced for regenerated detectors, for which the intercept assumes large and negative values with very high uncertainty, suggesting increased instability at high doses.

**Figure 5:**
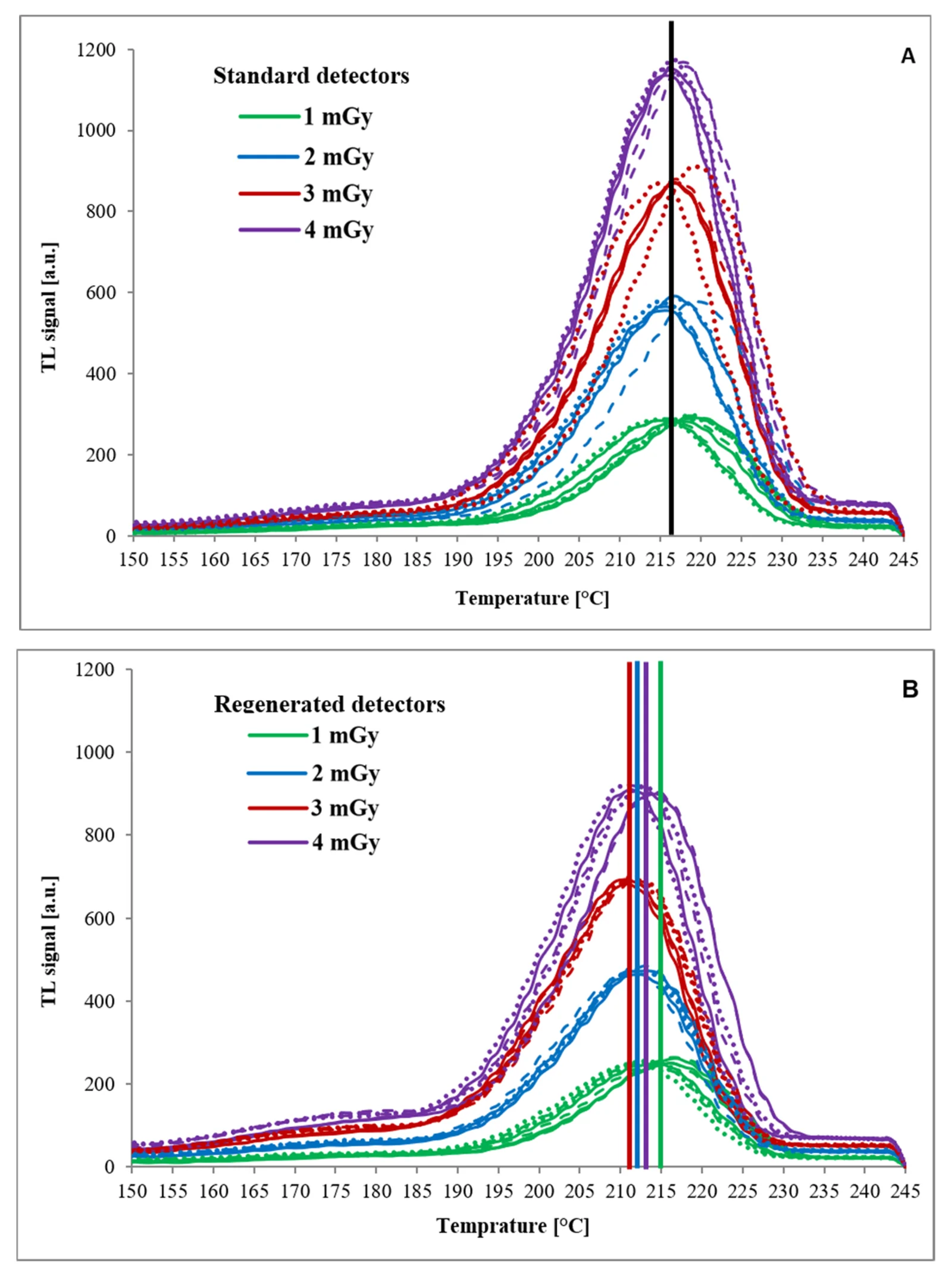
Thermoluminescence glow curves of standard (panel (**A**)) and regenerated (panel (**B**)) detectors in the 1–4 mGy range. The color of each curve corresponds to the irradiation dose, while the line style represents consecutive measurement series.

**Figure 6:**
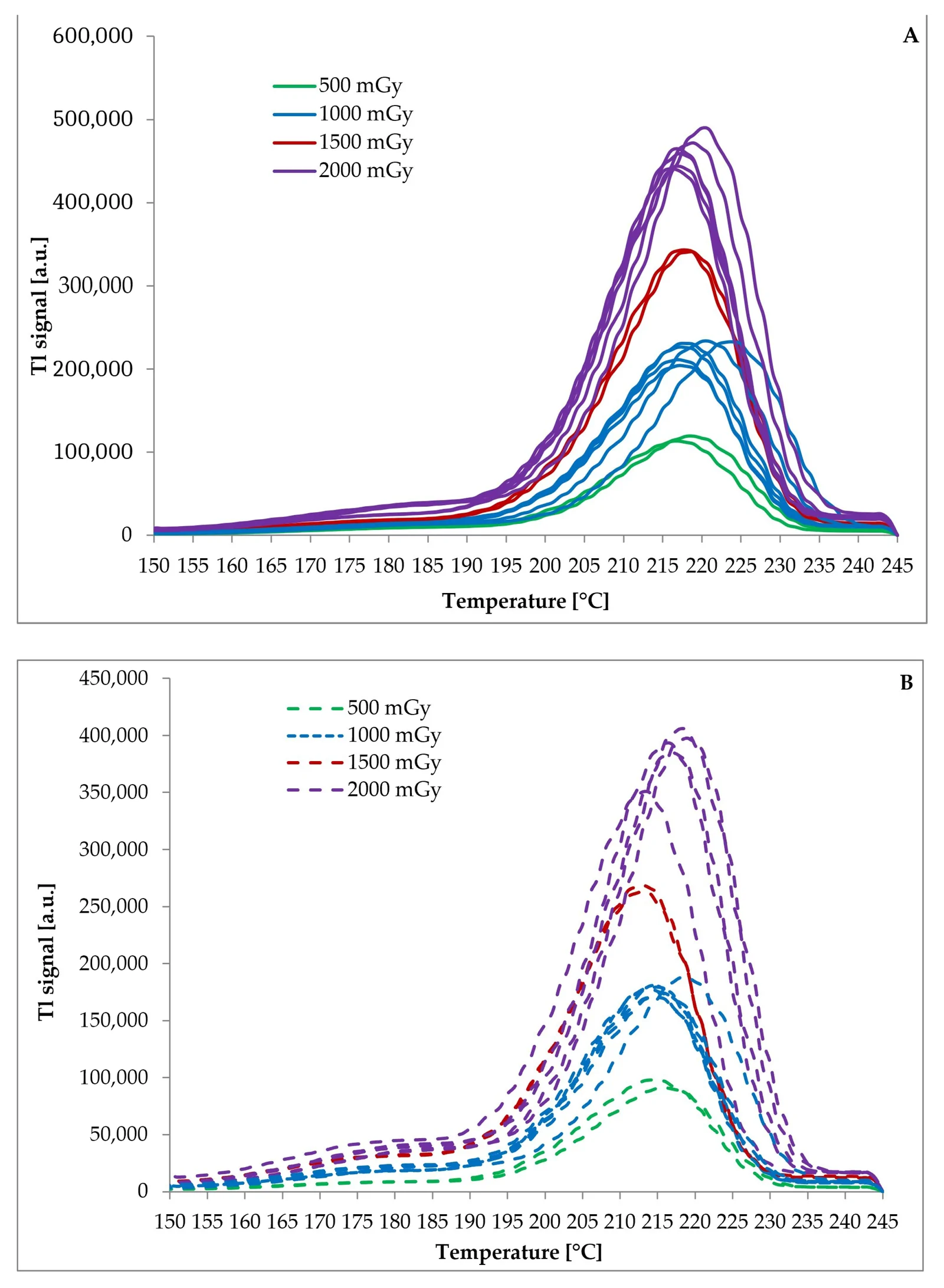
Thermoluminescence glow curves of standard (panel (**A**)) and regenerated (panel (**B**)) detectors in the 0.5–2 Gy range. The color of each curve corresponds to the irradiation dose, while the line style represents consecutive measurement series.

Additionally, Figure 5 and Figure 6 present the thermoluminescence glow curves of standard and regenerated detectors for both dose ranges. The color of each curve corresponds to the irradiation dose. The vertical lines indicate the average position of the amplitude, with their colors also corresponding to dose. For standard detectors in the 1–4 mGy range, the peak position remained unchanged with dose; therefore, a common mean maximum position was determined.

The thermoluminescence (TL) glow curves reveal distinct differences between standard and regenerated detectors, as well as between the low- and high-dose ranges.

In the low-dose range (1–4 mGy, Figure 5), regenerated detectors exhibit a higher degree of convergence of glow curves within groups corresponding to the same dose. At the same time, a more pronounced increase in the amplitude of low-temperature peaks is observed compared to standard detectors. In contrast, standard detectors show more consistent peak shapes with less pronounced low-temperature contributions. Importantly, the position of the dosimetric peak maximum remains essentially unchanged with increasing dose for both detector types, indicating stable trapping conditions in this dose range.

In the high-dose range (0.5–2 Gy, Figure 6), the behavior differs markedly. Standard detectors demonstrate better convergence of glow curves within dose groups, indicating greater stability. In contrast, regenerated detectors show increased dispersion and a enhancement of low-temperature peaks. Additionally, for doses up to approximately 1.5 Gy, the position of the dosimetric peak maximum for regenerated detectors shifts toward lower temperatures compared to standard detectors, suggesting modifications in trap structure and recombination processes induced by regeneration and high-dose exposure.

The quantitative values of the mean glow-peak temperature, standard deviation, and range presented earlier in Figure 4 and Figure 5 are shown in Table 5.

The mean dosimetric peak temperature was systematically lower for regenerated detectors than for standard detectors at every dose investigated (by 1.3–5.5 °C, mean ~3.5 °C), while within-batch variability for each detector type remained on the order of 1–2 °C (SD), consistent with typical instrumental scatter reported for TL peak-position measurements for the used reader. Because the shift is consistent in direction and magnitude across the dose range rather than randomly distributed, it exceeds expected instrumental variability and is attributed to genuine differences in trap distribution between detector types, as discussed later.

Summarizing, these observations confirm that while regenerated detectors can exhibit good stability at low doses, their glow-curve shape and peak position become more sensitive to dose and measurement conditions at higher dose levels.

### 3.7. Fading

To evaluate the fading effect, defined as the decay of the thermoluminescent signal over time, the detector response was analyzed as a function of the time elapsed between irradiation and readout. This effect was assessed for two dose levels, 0.25 Gy and 0.3 Gy (two different radiation sources) and 2 Gy, over a time interval ranging from 1 day up to 3 months after irradiation. All measurements were performed in ANALYSER mode to ensure stable and reliable readout conditions.

**Figure 7:**
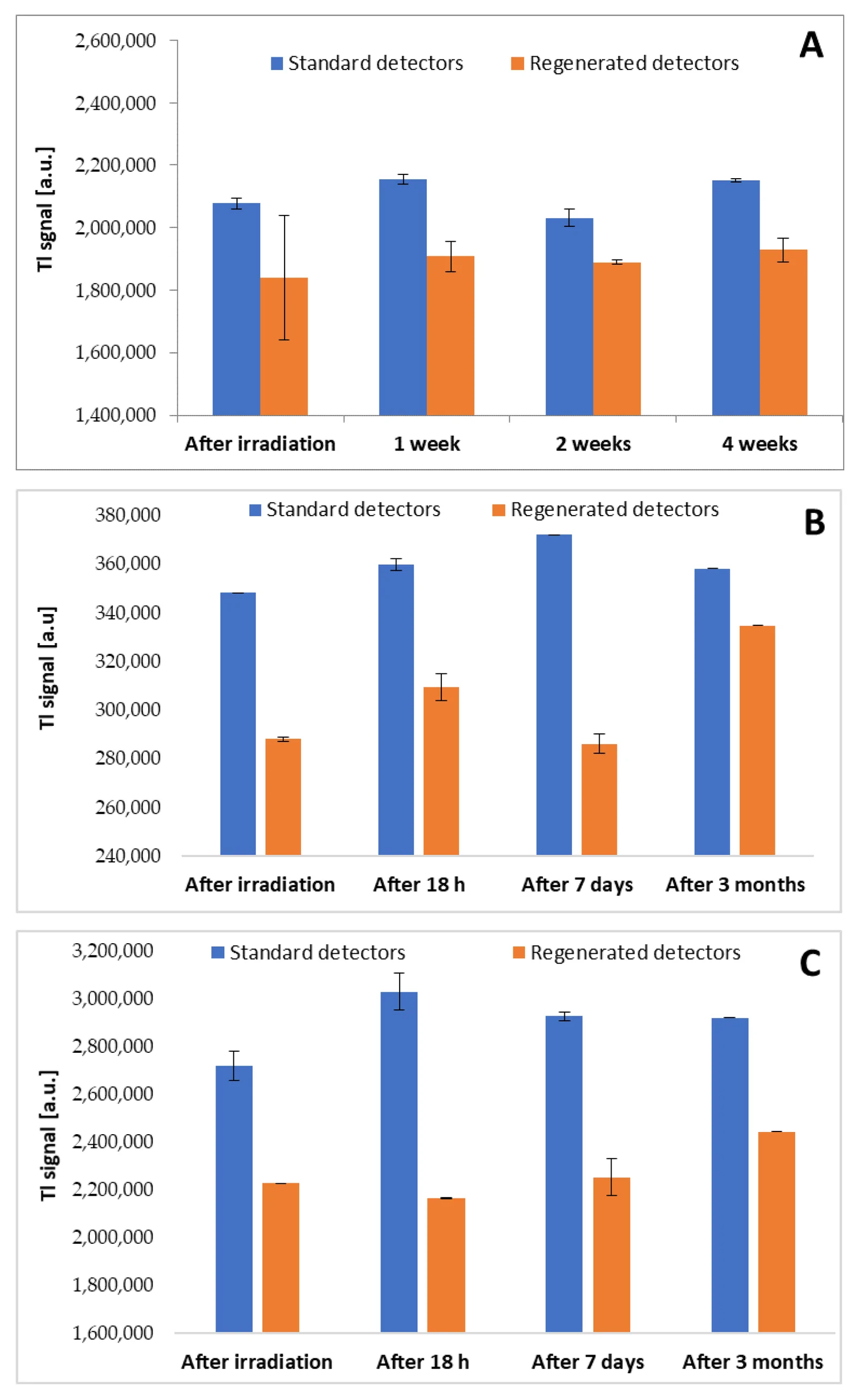
Fading of the thermoluminescent signal for standard and regenerated detectors at doses of 0.3 Gy (Cs-137)—panel (**A**), 0.25 Gy (Sr-90)—panel (**B**), and 2 Gy (Sr-90)—panel (**C**). The plots show the correcterd by IRF TL signal as a function of time elapsed between irradiation and readout, ranging from immediately after irradiation up to 3 months. Values represent median responses of detector groups.

The readings were normalized using IRF values determined in the reference measurements performed immediately prior to the fading test. Figure 7 presents the median response of detector groups as a function of time, respectively for 0.25 Gy, 0.3 Gy and 2 Gy.

The fading results for the three irradiation conditions (0.3 Gy—Cs-137 and 2 Gy and 0.25 Gy—Sr-90) show that the detector response does not exhibit the typical monotonic decrease over time. Instead, relatively stable values or even slight increases are observed over the period investigated.

For the 0.3 Gy dose (Cs-137), the normalized signal remains nearly constant over time, with only minor fluctuations within the measurement uncertainty. Similar behavior is observed for higher doses from the Sr-90 source. For the 2 Gy dose, the signal initially increases after irradiation (visible after ~18 h), followed by a slight decrease and subsequent stabilization over longer timescales (up to 3 months). For the 0.25 Gy dose, fluctuations are also observed, with no clear systematic decay trend, particularly for regenerated detectors, where a noticeable increase in response appears after longer storage times.

Such behavior indicates that the fading process in MCP-N detectors is not dominated by simple signal loss from unstable traps. Instead, competing processes may occur, including redistribution of charge carriers between trapping centers, partial retrapping, and thermal stabilization of deeper traps. These effects can lead to apparent signal recovery or stabilization over time, especially when the readout is based on the integral of the glow curve rather than a single peak.

Additionally, the absence of high fading may be attributed to the dominance of relatively stable trapping centers at the investigated dose levels, particularly in ANALYSER mode, where the full glow curve is considered. This results in improved long-term signal stability compared to what would be expected from single-peak analysis.

Overall, the results suggest that, within the studied time range, MCP-N detectors demonstrate good temporal stability of the TL signal, and that fading effects are either weak or partially compensated by internal processes within the detector material.

## 4. Discussion

Measurements were performed in two readout modes, READER and ANALYSER. The parallel use of two modes throughout this study was not a source of confounding but a deliberate methodological control. Because both modes were applied to every detector type at every dose level, the two variables under investigation—detector regeneration and readout procedure—can be evaluated separately rather than mixed together. A difference between standard and regenerated detectors that persists across both readout modes can be attributed to the detectors themselves, independent of how the signal is acquired. Conversely, a difference that appears in only one mode indicates that it originates from the readout procedure rather than from the regeneration process. This parallel design therefore strengthens, rather than weakens, the interpretation of the observed detector-type differences, and is consistent with established practice for MCP-N detector characterization [3,29,30,31]. The comparison indicates that the readout procedure is a key factor influencing detector performance, particularly at elevated doses. The reduced stability observed in READER mode is consistent with the known sensitivity of TL readout to glow curve shape variations and limitations of simplified integrating approaches [34]. At higher doses, where low-temperature peaks become more pronounced and the total signal increases, even small variations in the signal structure may lead to fluctuations in the integrated signal. In contrast, ANALYSER mode, which accounts for the full glow-curve shape, is less affected by such variations and therefore provides improved stability across the investigated dose range [34,35].

The observed differences between standard and regenerated detectors can be rationalized in terms of defect chemistry inherited from the regeneration process itself. The removal of copper ions during acid washing is very effective: a blue coloration of the solution is observed, without dissolution or decomposition of the underlying material, indicating that the copper ions are distributed within the detector structure at interstitial positions in the LiF crystal lattice rather than being incorporated into the host structure itself. This selective, non-destructive removal of Cu^2+^ does not, however, reset structural defects such as dislocations or vacancy aggregates already present in the recovered host lattice. Because Mg-, Cu-, and P-related defect complexes form through diffusion-controlled processes during melt solidification, combining independently re-doped recovered powder with virgin doped powder at a 1:1 ratio does not guarantee compositional homogeneity equivalent to a single-source melt; this is consistent with the broader Mg doping range measured for regenerated detectors (0.2–0.3 mol%) compared to the fixed value in standard detectors (0.2 mol%). A wider distribution of Mg-related complex sizes would be expected to broaden and shift the dosimetric peak, as observed, and to have a disproportionate effect at higher doses, where a wider range of trap depths—including those associated with inherited or regeneration-induced defects—becomes populated. This is consistent with the good agreement between detector types at low doses and the growing deviation observed at 0.5–2.0 Gy.

Although the regeneration procedure applied in the present study differs from conventional thermal recovery protocols reported in the literature, and to the best of our knowledge no published study has investigated post-production regeneration of LiF:Mg,Cu,P comparable to that employed here, previous experimental studies on thermally induced changes in trapping and recombination centres provide a useful framework for interpreting the present results [27,36,37]. This gap is consistent with the broader phosphor-recycling literature, which has generally focused on the recovery and purification of luminescent raw materials from production or lamp waste [38,39] rather than on the effects of such processes on the luminescence properties themselves. An exception is the study reported in [40], which reported measurable effects of milling and thermal regeneration on the luminescence properties of Eu^2+^- and Dy^3+^-doped strontium aluminate phosphors, including a slight shift to deeper levels in the estimated trap depths.

The TL response of LiF:Mg,Cu,P strongly depends on the configuration of trapping and recombination centres associated with Mg, Cu and P dopants. Experimental studies have shown that thermal treatment modifies the population and spatial arrangement of Mg-related defect complexes, thereby affecting the intensities of individual glow peaks [41]. Similarly, systematic investigations of detectors with different Mg, Cu and P concentrations demonstrated that both the glow-curve structure and detector sensitivity are strongly dependent on dopant composition and thermal history [42,43,44]. In addition, EXAFS measurements indicated that thermal treatment may alter the oxidation state and local coordination of Cu ions, suggesting that modifications of recombination centres also contribute to changes in detector sensitivity [45].

Further evidence for thermally induced changes in recombination centres has been reported, identifying two principal luminescence components in LiF:Mg,Cu,P and demonstrating that one type of recombination centre disappears above approximately 240 °C but can be partially restored during cooling, whereas the other undergoes conversion into a different configuration [46]. Importantly, it has been concluded that the microscopic origin of sensitivity loss, sensitivity recovery and glow-curve modifications remains unresolved, indicating that several defect-related processes probably occur simultaneously.

Additional support for defect reorganization rather than irreversible degradation is provided by evidence that optimized annealing after severe sensitivity loss induced by high-dose irradiation and high-temperature readout enabled recovery of MCP detector sensitivity [27]. These results indicate that the defect structure responsible for the high sensitivity of LiF:Mg,Cu,P can be reconstructed under appropriate thermal conditions.

The present results are consistent with these experimental observations. The preserved linear dose response, high repeatability and comparable fading indicate that the regenerated material retains the fundamental dosimetric properties of conventional MCP-N detectors, while the reduced sensitivity, enhancement of the low-temperature peaks, and the shift and broadening of the main dosimetric peak are consistent with the redistribution and possible partial reconstruction of Mg-, Cu- and P-related trapping and recombination complexes induced by the regeneration process, rather than a new luminescence mechanism [27,41,42,46]. Nevertheless, this interpretation should be regarded as physically plausible rather than conclusive, as no experimental studies have yet investigated defect evolution during post-production regeneration comparable to that employed in the present work.

The temporal evolution of the detector response reflects typical conditioning effects observed in thermoluminescent materials. The improvement in repeatability after successive irradiation–readout cycles suggest stabilization of charge carrier populations in trapping centers. Initially unstable shallow traps are progressively depleted or reorganized, and the response becomes dominated by deeper and thermally stable traps [47,48]. This effect is particularly relevant for low doses, where shallow trap contributions are relatively significant and strongly influence signal variability.

A comparison of the repeatability parameter (R) and IRF values show that, excluding READER mode at high doses, higher dose levels generally provide better reproducibility. Repeatability improved with increasing dose in the low-dose range, suggesting a reduced influence of signal-to-noise limitations and shallow-trap instability.

Regarding detector type, standard detectors generally exhibit slightly better reproducibility, particularly under unstable conditions such as low doses and READER mode operation. However, after stabilization, regenerated detectors achieve reproducibility comparable to that of standard detectors, indicating that regeneration does not considerably impair detector performance.

Differences between standard and regenerated detectors can be primarily linked to modifications in trap distribution introduced during the regeneration process. Regenerated detectors tend to exhibit enhanced low-temperature peaks and slightly shifted dosimetric peak positions, indicating changes in trapping and recombination mechanisms [23,34]. These effects are more pronounced at higher doses, where additional trapping centres may become involved. As a result, regenerated detectors show slightly higher variability under unstable conditions, although their performance becomes comparable to standard detectors once stabilization is achieved [1,49].

Both detector types show good linearity over the investigated dose ranges. In the low-dose range, the fitted parameters indicate highly reliable linear behavior, whereas increased uncertainties at higher doses suggest the onset of non-linear processes. At higher doses (0.5–2.0 Gy), uncertainties increase, particularly for regenerated detectors, where large and negative intercept values are observed. This suggests deviations from ideal linearity, likely due to changes in trapping mechanisms, such as the involvement of deeper traps or recombination centers at higher irradiation levels [19,25].

Analysis of thermoluminescence glow curves provides further insight into these mechanisms. The stability of the dosimetric peak position at low doses indicates unchanged trapping conditions, whereas the observed peak shifts and broadening at higher doses for regenerated detectors suggest alterations in trap distribution and charge carrier dynamics. Such dose-dependent changes in glow-curve shape are well documented and reflect the complex nature of thermoluminescence processes, which involve multiple trapping and recombination centers [11].

The fading study indicates that the thermoluminescent signal remains relatively stable over time and does not exhibit a simple monotonic decay. This behavior is consistent with literature reports showing that LiF:Mg,Cu,P detectors exhibit low fading, with signal variations typically remaining within a few percent over extended storage periods [2,20,25]. The absence of clear signal decay may be explained by competing processes such as retrapping and redistribution of charge carriers between trapping centres, which can partially compensate for loss from shallow traps [20]. As a result, apparent signal stabilization or even slight increases may be observed over time. The results of the research on fading are consistent with the literature data [12,25,26].

The present results demonstrate that the choice of readout mode is a critical factor in determining detector performance. While both standard and regenerated detectors provide reliable measurements in ANALYSER mode, the instability observed in READER mode at higher doses limits its applicability in this range. Further results suggest that mechanical filtration may reduce the influence of low-temperature emission and improve measurement stability, which should be investigated in future studies.

Overall, the results demonstrate that regenerated MCP-N detectors retain dosimetric properties comparable to those of standard detectors, particularly after stabilization and when evaluated in ANALYSER mode, supporting their potential applicability in routine thermoluminescent dosimetry.

## 5. Conclusions

The performed analysis confirms that both standard and regenerated MCP-N thermoluminescent detectors exhibit good dosimetric properties, including high repeatability, stable IRF values, linear dose response, and comparable fading behavior.

Detector response was found to depend on temporal stabilization processes, with improved repeatability observed after successive irradiation–readout cycles. Although standard detectors generally exhibit slightly better performance under non-optimal conditions, regenerated detectors achieve comparable properties after stabilization, demonstrating their practical usability.

The results highlight that the readout mode is a key factor governing detector performance. The ANALYSER mode ensures stable and reliable response over a wide dose range, whereas the READER mode becomes increasingly unstable at higher doses, limiting its applicability without additional optimization like, e.g., additional filtration of the signal.

Standard detectors are characterized by higher sensitivity, while regenerated detectors show repeatability comparable to standard detectors after stabilization. Homogeneity of regenerated detectors was mode-dependent: lower than standard detectors in READER mode, and only comparable to standard detectors in ANALYSER mode, rather than improved overall. Despite reduced sensitivity, regenerated detectors provide consistent and reliable measurements after proper calibration.

Both detector types show good linearity within the investigated dose ranges. However, deviations from ideal behavior at higher doses, together with changes in glow-curve shape and peak position, indicate modifications in trapping structure and the involvement of additional recombination mechanisms, particularly for regenerated detectors.

Fading studies indicate relatively stable signal levels over time, which can be explained by charge redistribution and retrapping effects.

It should be noted that the regenerated detectors examined in this work were produced from recycled LiF originating from production waste, not from detectors that had undergone dosimetric use and the associated sensitivity degradation from repeated irradiation–annealing cycles. The present results therefore demonstrate the feasibility of recovering usable detector material from manufacturing waste, but do not establish whether the proposed approach can restore the performance of aged, dosimetrically degraded detectors. These are related but distinct problems and extending this approach to used detectors would require separate validation.

Overall, the results demonstrate that regenerated MCP-N detectors can serve as a viable and more sustainable alternative to standard detectors. The findings also emphasize the importance of optimizing readout conditions, particularly using ANALYSER mode and potential application of mechanical filtration, to ensure reliable dosimetric performance across a wide range of doses.

Standard detectors demonstrate favorable performance at higher dose levels, which may suggest potential suitability for radiotherapy applications; however, since these results are based on a single measurement series, this observation should be considered preliminary and would require confirmation through additional independent measurements before firmer conclusions regarding linearity or underlying trapping/recombination mechanisms can be drawn. Regenerated detectors provided a stable response in the low-dose range, supporting their potential suitability for radiological protection applications.

## Figures and Tables

**Table 1:** Division of detectors into groups according to the type and mode of reading together with the number of groups.

Readout Mode	Type of Detectors	
	Standard	Regenerated
READER	8	9
ANALYSER	9	9

**Table 2:** Batch homogeneity.

Readout Mode	Type of Detectors	
	Standard	Regenerated
READER	8.41	20.27
ANALYSER	8.06	7.94

**Table 3:** Dependence of reading reproducibility on the number of series and dose.

	READER Mode			
	Standard detectors		Regenerated detectors	
	Dose 1 mGy			
	1–18	16–18	1–18	16–18
Median ± Range [%]	76.2 ± 9.4	97.5 ± 2.7	73.9 ± 9.9	98.1 ± 1.5
Relative uncertainty [%]	12.3	2.7	13.4	1.6
	Dose 4 mGy			
Median ± Range [%]	97.9 ± 2.9		99.1 ± 1.1	
Relative uncertainty [%]	3.0		1.1	
	Dose 240 mGy			
Median ± Range [%]	59.3 ± 76.9		74.5 ± 114.4	
Relative uncertainty [%]	129.67		153.4	
	ANALYSER mode			
	Standard detectors		Regenerated detectors	
	Dose 1 mGy			
	1–18	16–18	1–18	16–18
Median ± Range [%]	80.7 ± 7.9	97.9 ± 4.2	76.9 ± 11.4	95.6 ± 3.7
Relative uncertainty [%]	9.8	4.3	14.9	3.9
	Dose 4 mGy			
Median ± Range [%]	97.9 ± 2.4		99.1 ± 1.6	
Relative uncertainty [%]	2.4		1.6	
	Dose 240 mGy			
Median ± Range [%]	97.2 ± 5.1		92.9 ± 7.9	
Relative uncertainty [%]	5.2		8.5	

**Table 4:** Stability of the readings for a dose of 1 Gy and 2 Gy.

Dose	Standard Detectors		Regenerated Detectors	
	READER Mode	ANALYSER Mode	READER Mode	ANALYSER Mode
1 Gy	81.3	90.7	96.4	94.9
2 Gy	76.4	96.1	−16.7	90.0

**Table 5:** Mean glow-peak temperature (±SD) and range (min–max) for standard and regenerated detectors as a function of dose, corresponding to the data presented in Figure 4 and Figure 5.

Dose	Standard Detectors		Regenerated Detectors	
	Mean ± SD	Min–Max	Mean ± SD	Min–Max
1 mGy	217.2 ± 1.6	215–219	214.7 ± 1.8	212–217
2 mGy	216.5 ± 1.6	215–219	212.0 ± 0.9	211–213
3 mGy	216.5 ± 1.3	214–217	211.3 ± 0.8	210–212
4 mGy	217.2 ± 1.4	216–219	212.5 ± 1.4	211–214
0.5 Gy	218.0 ± 1.5	217–219	214.5 ± 0.8	214–215
1.0 Gy	219.0 ± 2.9	217–224	215.3 ± 1.9	214–219
1.5 Gy	218.5 ± 0.8	218–219	213.0 ± 0.0	213
2.0 Gy	217.7 ± 1.6	216–220	216.4 ± 2.1	213–218

## Data Availability

Data available on request.
